# A novel *PSEN2* mutation in amnestic early-onset Alzheimer's disease (EOAD): A familial case series

**DOI:** 10.1177/25424823251348676

**Published:** 2025-06-25

**Authors:** Carl Froilan D Leochico, Ekaterina Rogaeva, Ljubica Zotovic, Ana Luiza Pinto Oliveira, Tina Le, Amit Singnurkar, Mario Masellis, Sara B Mitchell

**Affiliations:** 1 Department of Psychiatry, Sunnybrook Health Sciences Centre, University of Toronto, Toronto, Canada; 2 Division of Neurology, Department of Medicine, University of Toronto, Toronto, Canada; 3 College of Medicine and Philippine General Hospital, University of the Philippines Manila, Manila, Philippines; 4 Department of Physical Medicine and Rehabilitation, St Luke's Medical Center, Global City and Quezon City, Philippines; 5 Azrieli Brain Medicine Fellowship Program, University of Toronto, Toronto, Canada; 6 Tanz Centre for Research in Neurodegenerative Disease, University of Toronto, Toronto, Canada; 7 Sunnybrook Research Institute, Sunnybrook Health Sciences Centre, Toronto, Canada; 8 Department of Medical Imaging, Sunnybrook Health Sciences Centre, University of Toronto, Toronto, Canada; 9 Neurology Quality and Innovation Lab (NQIL), University of Toronto, Toronto, Canada

**Keywords:** Alzheimer's disease, dementia, early-onset alzheimer's disease, familial alzheimer's disease, genetics, mutation

## Abstract

Familial early-onset Alzheimer's disease (EOAD) is a rare form of dementia often caused by autosomal dominant mutations in *APP*, *PSEN1,* or *PSEN2*. We report a novel *PSEN2* missense variant (c.359T > G, p.Ile120Ser) that has been detected in four siblings; three of whom are affected by predominantly amnestic EOAD or mild cognitive impairment in their fifties (supported by neuroimaging biomarkers), while the youngest sibling is currently asymptomatic at age 50. Two of the siblings were also heterozygous for a variant in *PSEN1* (c.118_120del, p.Asp40del). Between the two genes, the *PSEN2* variant was deemed to be likely pathogenic based on segregation with EOAD, imaging biomarker analyses, and bioinformatic analyses. Reporting genetic findings in familial EOAD cases can help in classifying their pathogenic significance and improving genetic conceptualization within Alzheimer's disease.

## Introduction

The World Health Organization estimates that there are currently 55 million people with dementia, and 10 million new cases are reported annually worldwide with a growing prevalence.^1^ Alzheimer's disease (AD) is the most common form of dementia, contributing to 60–80% of cases.^2^ Advanced age is the strongest AD risk factor. AD with symptom onset before age 65 is typically defined as early-onset AD (EOAD), which accounts for 5–10% of all AD cases.^3^ While approximately one in 10 cases has an autosomal dominant segregation pattern, only a small number of these are caused by rare mutations (minor allele frequency <0.001) in at least one of the three causal EOAD genes, including amyloid precursor protein (*APP*), presenilin 1 (*PSEN1*), or presenilin 2 (*PSEN2*), leaving the majority of cases unexplained.^4–6^ It is also often challenging to interpret the pathogenic significance of genetic variations in the causal EOAD genes, as evident from the Alzforum Mutations database (https://www.alzforum.org/mutations). Indeed, numerous unclassified variants in these genes lack sufficient proof for their pathogenic significance due to unreported cases with detected mutations, as well as a lack of either segregation with EOAD in at least one large family or recurrence of the mutation in several unrelated EOAD cases.^7^ Hence, to contribute to the classification of rare variants, we report the clinical and genetic findings in a Canadian family of Western European descent affected by EOAD.

## Case presentation

**Figure 1.**
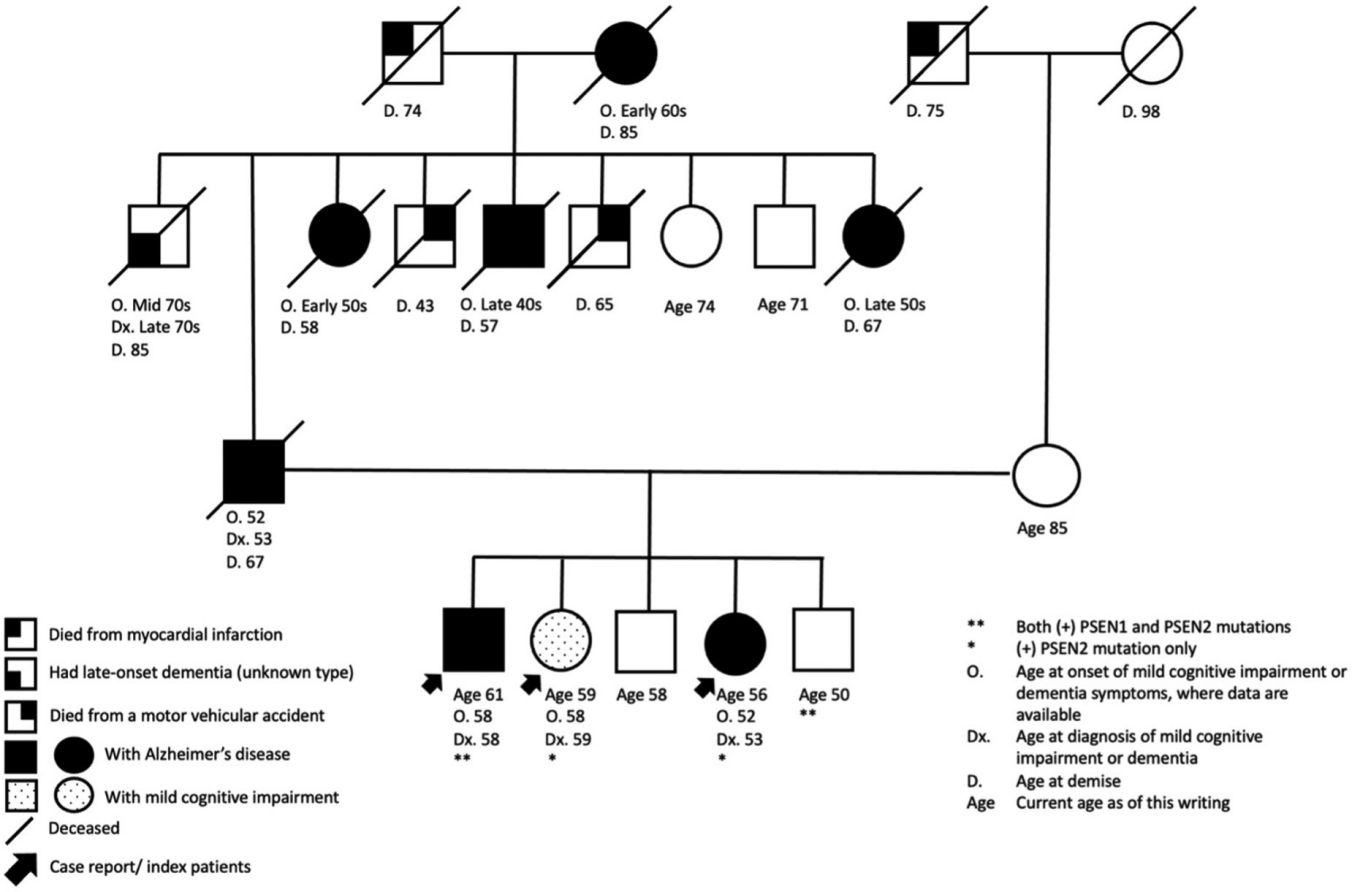
Family tree demonstrating the patients’ significant family history of dementia.

Patients A, B, and C are full siblings who presented to our cognitive neurology clinic several years apart. They have two other siblings who remain asymptomatic and have a significant family history of dementia (Figure 1). Their paternal grandmother had cognitive impairment with onset in her early 50s, while their father was diagnosed with EOAD at age 53 and three of her father's eight siblings had EOAD with onset in their 40s or 50s. We first saw Patient A (the fourth oldest sibling), a 53-year-old female with 16 years of education who presented with a 1.5-year history of gradual-onset cognitive decline in the context of a major depressive episode (managed with fluoxetine) and life stressors, including relationship and financial difficulties. She went on leave from her managerial-level job, which she found overwhelming. The family noted she had become more reliant on calendars to remember appointments and that she misplaced common items, repeated questions and statements, left appliances on, and had trouble remembering how to use appliances. Word-finding worsened over time, although comprehension remained intact. She developed disinhibited behaviors along with executive dysfunction manifesting as decision-making challenges and impulsive spending. There were no parkinsonian features or other motor manifestations. She was independent in basic activities of daily living (ADLs), although there was some dressing apraxia and she needed assistance with most instrumental ADLs (iADLs) like meal preparation, shopping, and finances.

On initial evaluation, she was alert and oriented to three spheres. The neurologic examination revealed orobuccal and bilateral limb apraxia. On the Behavioral Neurology Assessment – Short Form (BNA-SF),^8^ she scored 58/114 (normal: ≥ 82) with impairment on all domains (attention, memory, language, visuospatial, and executive). A provisional diagnosis of EOAD was established and the patient was started on donepezil.

**Figure 2.**
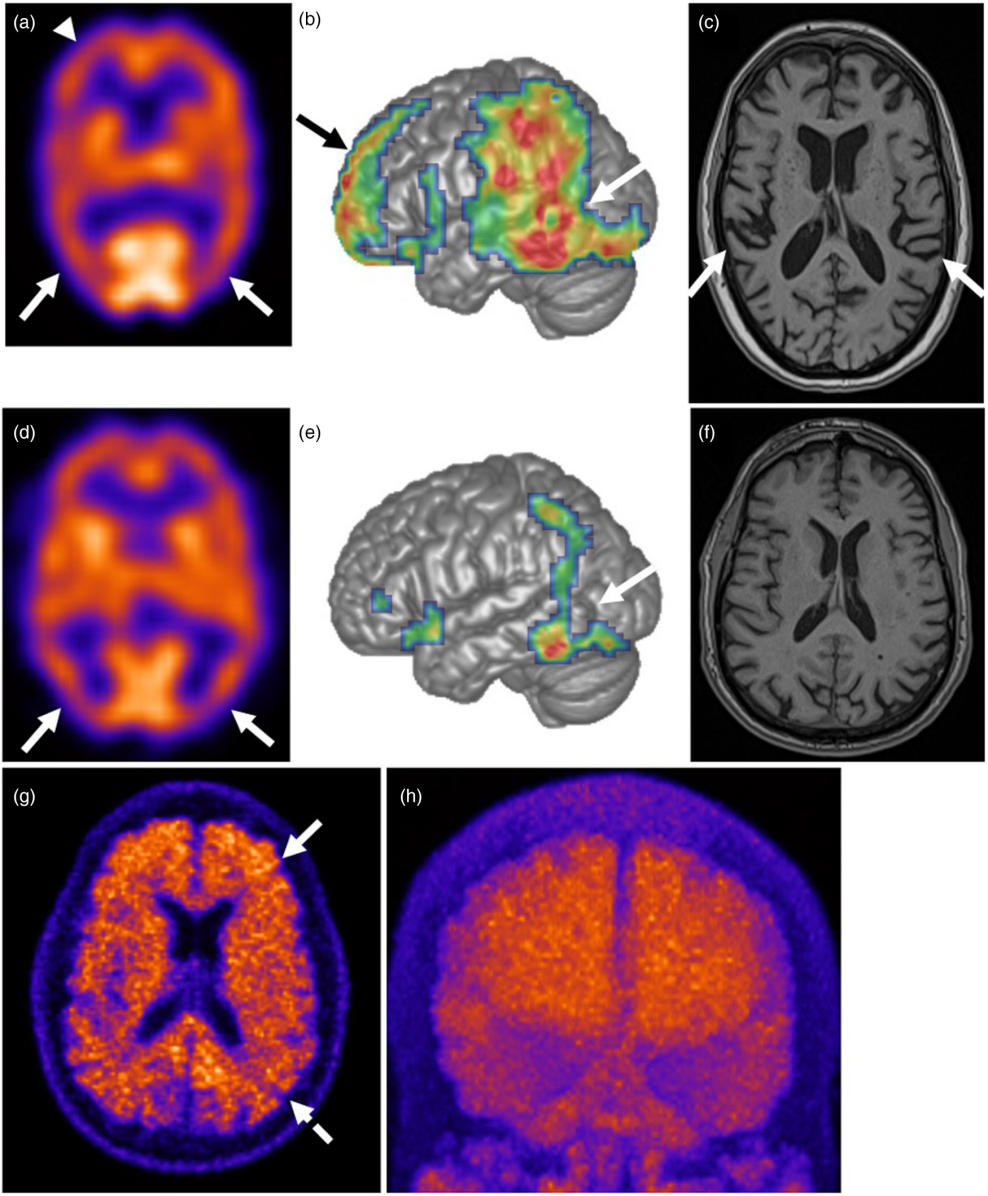
**Patient A:** Selected transaxial slice of a ^99m^Tc-ethyl cysteinate dimer (ECD) brain perfusion study (a) shows more extensive areas of moderately reduced perfusion to the parietotemporal regions (white arrows) and reduced uptake in the prefrontal region (arrowhead) with relative sparing of the somesthetic and motor cortices and occipital lobes, also shown on statistical parametric mapping (SPM) (white and black arrows, respectively) (b). MRI T1-weighted transaxial images (c) show mild temporoparietal lobe-predominant volume loss (white arrows). **Patient B:** Selected transaxial slice of a ^99m^Tc-ECD brain perfusion study (d) shows mildly reduced perfusion to the parietotemporal association regions (white arrows), also demonstrated on SPM (white arrow) (e). MRI T1-weighted transaxial images (f) show no regional or global cortical atrophy. On ^18^F-florbetaben (Neuraceq) transaxial PET imaging (g), there is a diffuse tracer localization that shows homogeneous uptake across both the white and gray matter in the frontal and parietal lobes (white solid and dashed arrows, respectively), indicating high amyloid-β deposition. Anterior volumetric maximum intensity projection (h) shows the global extent of tracer uptake seen throughout the cerebral gray and white matter.

**Figure 3.**
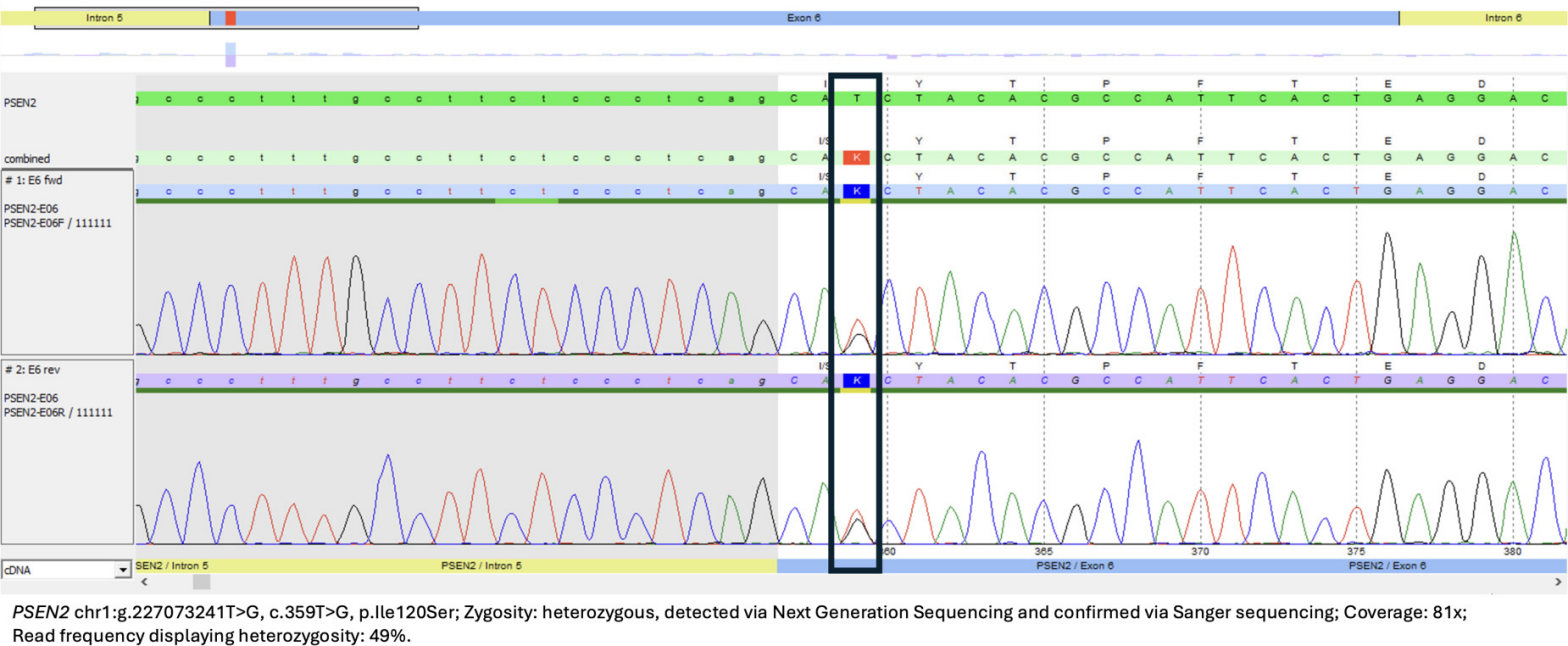
Sanger sequencing chromatogram confirming the presence of the p.Ile120Ser mutation in *PSEN2* (patient A).

Blood test results for reversible causes of cognitive impairment were unremarkable. A brain single-photon emission computed tomography (SPECT) showed reduced tracer uptake in bilateral parietotemporal and prefrontal regions with relative sparing of the primary somesthetic and motor cortices (Figure 2(a) and (b)), while brain magnetic resonance imaging (MRI) revealed mild temporoparietal-predominant atrophy (Figure 2(c)). Genetic testing was performed through LifeLabs Genetics and CENTOGENE, wherein Next Generation Sequencing technology was used to cover the coding regions of the various dementia-related genes (Supplemental Material 1), including 10 bp of intronic regions flanking each exon. The patient was found to be heterozygous for a c.359T > G variant in *PSEN2* predicted to result in an amino acid substitution (p.Ile120Ser), which was classified as a variant of uncertain significance and, to our knowledge, has not been reported in the literature or in a large population database (http://gnomad.broadinstitute.org). The Sanger sequencing chromatogram is shown in Figure 3, confirming the presence of the p.Ile120Ser mutation in *PSEN2*. The rest of the dementia genetic panel was negative, including *APOE4*.

Over the next two years, Patient A displayed progressive cognitive and functional deterioration, with delusions and hallucinations eventually requiring admission to a long-term care facility.

Patient B, the oldest sibling, was next seen in our clinic a year after Patient A's initial visit. He was a 58-year-old male with over 18 years of education and well-controlled obstructive sleep apnea (OSA). He presented with a 2.5-year history of gradual-onset cognitive decline causing difficulty with his high-level job. He had difficulty transitioning to remote work during the onset of the COVID-19 pandemic, and his colleagues noted that he was unable to follow conversations due to slowed processing speed, impaired short-term memory, and word-finding difficulties. He endorsed symptoms of depression and anxiety (managed with vortioxetine). There were no associated parkinsonian or autonomic features. At the time of presentation, he was independent in ADLs and many iADLs, but he developed trouble following recipes, accessing email on his phone and managing his finances and eventually went on medical leave from work.

On initial evaluation, he was alert, ambulatory, well-kempt, and oriented to three spheres. He was euthymic with reactive affect. He had occasional word-finding pauses and semantic paraphasia. He was occasionally tangential but redirectable with normal thought content, insight, and judgment. His neurological examination revealed subtle right-sided ideomotor apraxia. On finger tapping, he had mild decrement and slowness accompanied by mirror movements. On the Montreal Cognitive Assessment (MoCA), he scored 23/30 (normal: ≥ 26), wherein he lost one point each on clock drawing and hand tapping and five points on delayed recall (Memory Index Score: 3/15).^9^ A provisional diagnosis of EOAD was established, and the patient was started on donepezil.

On neuropsychological assessment, the patient's general intellectual functioning was in the high average range with superior vocabulary knowledge, consistent with his educational and occupational expectations. In this context, his performance was suggestive of a predominantly amnestic EOAD, given his below-expectation scores across most verbal and visual memory tests and a pattern of weaker semantic relative to phonemic fluency.

Blood tests for reversible causes of dementia were unremarkable. A brain SPECT found minimal bilateral mesiotemporal and frontal hypoperfusion, suggestive of an anterior-posterior gradient (Figure 2(d) and (e)). A brain MRI did not show significant cortical atrophy (Figure 2(f)). An amyloid positron emission tomography (PET) revealed pronounced beta-amyloid deposition, with regional cortical tracer uptake (RCTU) scores of 3 in all regions and an overall brain amyloid plaque load (BAPL) score of 3 (Figure 2(g) and (h)).

On genetic testing through PreventionGenetics, Patient B was found to be heterozygous for the same *PSEN2* variant as Patient A (p.Ile120Ser). Additionally, he was heterozygous for a *PSEN1* variant defined as c.118_120del, which was predicted to result in an in-frame GAC-deletion(p.Asp40del) and previously reported in a patient with EOAD without further segregation study (ClinVar ID 1505666, https://www.ncbi.nlm.nih.gov/clinvar/).^10^ The familial-onset AD panel did not include *APOE* testing. Over the next two years, Patient B exhibited progressive cognitive and functional decline and was unable to return to work. His MoCA scores decreased to 21/30 at 1-year follow-up, 18/30 at 1.5 years, and 13/30 at 2 years.

Patient C, the second oldest sibling, presented to our clinic two years after Patient B's initial visit. She was a 59-year-old woman with >18 years of education and a 1-year history of gradual-onset cognitive decline. She was misplacing objects around the house, leaving appliances on, and getting lost more frequently while driving. Nonetheless, she remained independent in ADLs and iADLs. There were no behavioral or personality changes, but she was feeling sad and worried about her siblings’ medical conditions.

On the MoCA, she scored 25/30, losing 5 points on delayed recall (Memory Index Score: 5/15).^9^ The neurological examination was unremarkable. She was given a provisional diagnosis of mild cognitive impairment (MCI).

Blood tests for reversible causes of dementia were unremarkable. A brain MRI showed microangiopathic changes and mild generalized volume loss with no lobar predominance. On neuropsychological testing, she met criteria for amnestic MCI, demonstrating encoding and retention deficits in verbal and visual episodic memory and weaker semantic fluency compared to phonemic fluency. On genetic testing through Invitae, Patient C was found to be heterozygous for the same *PSEN2* variant as Patients A and B (p.Ile120Ser). No *APOE* testing was done, similar to Patient B. Meanwhile, their currently asymptomatic youngest sibling (age 50) was found to be a mutation carrier for *PSEN1* and *PSEN2*. The asymptomatic middle sibling has not undergone genetic testing. One of their unaffected paternal uncles who was 71 years old got tested and was negative for both mutations.

## Discussion

**Figure 4.**
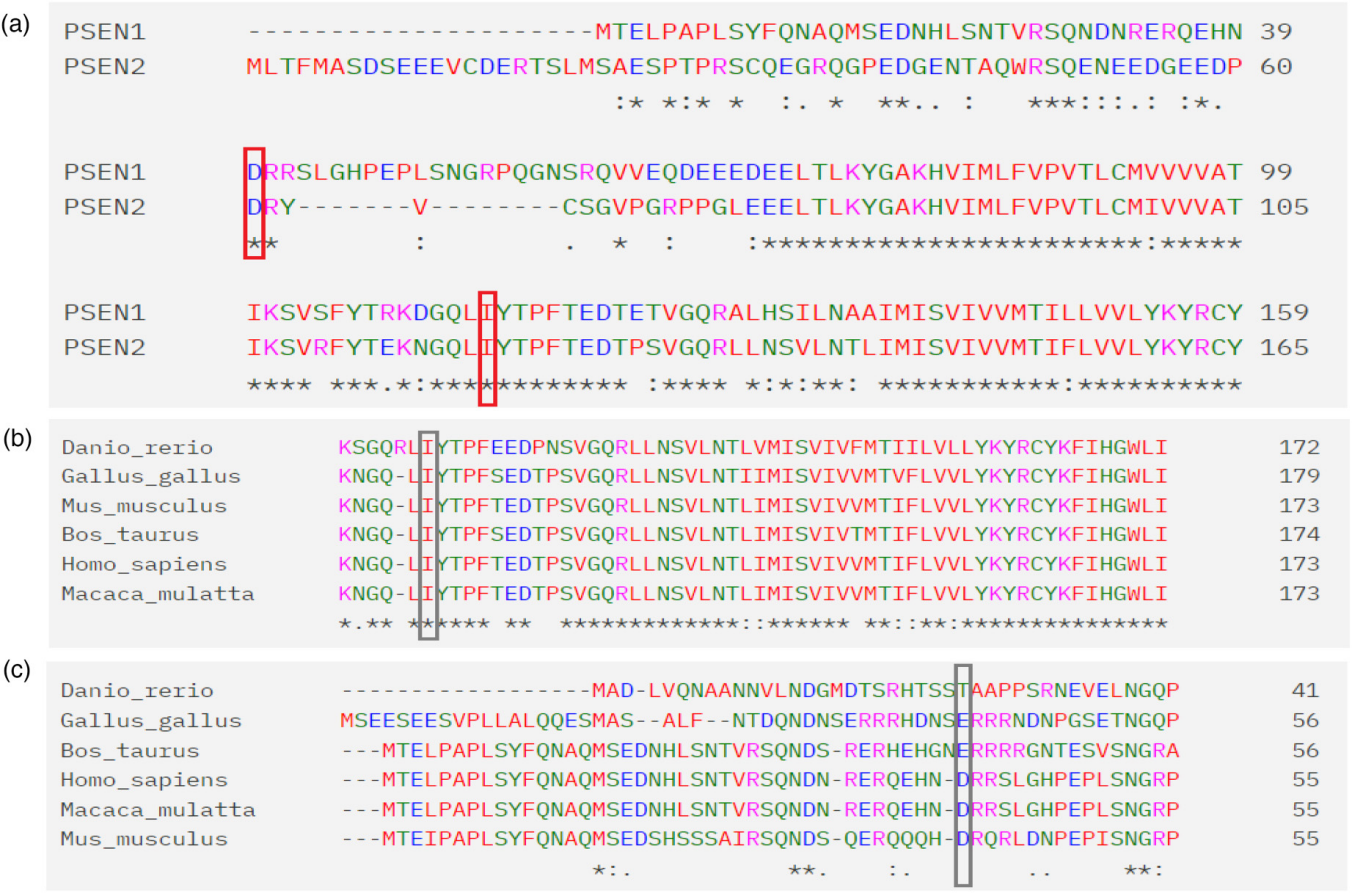
A partial alignment for conservation analysis of protein sequences between *PSEN1* and *PSEN2*, as well as across different species, highlighting the positions of the detected variants (p.Ile120Ser and p.Asp40del) with boxes: (a) Sequence comparison of *PSEN1* and *PSEN2* proteins; (b) Alignment of *PSEN2* protein sequences across species; and (c) Alignment of *PSEN1* protein sequences across species. The protein sequence alignments were prepared using Clustal Omega, and the sequences were obtained through https://www.uniprot.org/.

We present a familial case series of predominantly amnestic EOAD, supported by consistent structural and functional neuroimaging findings. Our data strongly support the pathogenic nature of the p.Ile120Ser substitution in *PSEN2*. First, it segregated with either EOAD/MCI in all three affected siblings. Second, bioinformatic analyses using In Silico, MutationTaster, and SIFT predict the damaging effect of the p.Ile120Ser mutation. Indeed, the p.Ile120Ser mutation has a very high Combined Annotation Dependent Depletion (CADD) score (Phred = 27.2), placing it in the top 0.1% of deleterious variants in the human genome. Third, the deleterious effect of p.Ile120Ser is supported by its extreme rarity, as it has not been reported in current genetic databases, including the global gnomAD database. Fourth, the mutation leads to the substantial replacement of a non-polar amino acid (Ile) with a polar amino acid (Ser) at a codon conserved between *PSEN1* and *PSEN2* proteins (Figure 4(a)), as well as across species during evolution (Figure 4(b) and (c)). Finally, the p.Ile120Ser mutation is located in the highly conserved HL-1 loop between Transmembrane domains 1 and 2, where four other *PSEN2* mutations have been reported (http://www.alzforum.org/mutations). These include non-classified mutations (p.Thr122Arg, p.Pro123Leu) and likely pathogenic mutations (p.Thr122Pro, p.Glu126Lys).

Notably, the homologous HL-1 loop in *PSEN1* contains up to 30 pathogenic or likely pathogenic mutations, indicating the functional significance of this region for γ-secretase activity (http://www.alzforum.org/mutations). Mutations in the HL-1 loop of *PSEN2* can disrupt γ-secretase function, leading to the altered cleavage of *APP* and subsequent accumulation of amyloid-β peptides, particularly the Aβ_42_ isoform, which is crucial in the formation of amyloid plaques in AD.^11^ The *PSEN2* T122P mutation has been identified in two (presumably unrelated) German individuals with probable EOAD (onset at ages 46 and 50) and a family history of dementia.^12,13^ It has also been reported in French families with probable EOAD, *APOE3/E4*, age of onset between 45–47 years, and disease duration of 2–7 years.^7^ Meanwhile, the other likely pathogenic *PSEN2* mutation, E126K, was identified in a congenitally deaf woman, who had increasing forgetfulness at age 48 and was severely demented, nonresponsive, and hospitalized since age 50.^14^ The variant was also detected in her mother, who might have developed symptoms earlier than age 59, was admitted to a nursing home by age 63, and died at age 72.^14^

In contrast, our study does not support the pathogenic significance of p.Asp40del in *PSEN1*, which is listed in the gnomAD database with a frequency of 0.0001. In our family, p.Asp40del did not segregate with EOAD, as it was detected in only one of the three affected siblings. Notably, the p.Asp40 codon is within a protein sequence that is not conserved between *PSEN2* and *PSEN1* (Figure 4(a)) or across species (Figure 4(b) and (c)). Furthermore, the CADD score for p.Asp40del is only 14.4, which does not support its pathogenic nature. In the mutation database (http://www.alzforum.org/mutations), p.Asp40del is classified as having “uncertain significance,” and its role as a disease risk modifier cannot be ruled out. However, in the family reported here, the carrier of both the p.Asp40del and p.Ile120Ser mutations (Patient B) developed the disease ∼5 years later than the carrier of only the p.Ile120Ser mutation (Patient A). Initially, the p.Asp40del mutation was reported in a patient with atypical AD, including symptoms of frontotemporal dementia.^10^ More recently, it was detected in a Belgian AD patient with decreased Aβ_42_/Aβ_43_ levels in cerebrospinal fluid, consistent with AD.^15^ However, functional studies of p.Asp40del have produced conflicting results.^16,17^

Furthermore, we have conducted a structure prediction for the p.Ile120Ser mutation in *PSEN2* and the p.Asp40del mutation in *PSEN1*, compared to the wildtype. Based on our analysis, neither mutation appears to alter the predicted 3D protein structure. However, the significance of this type of analysis is limited, as mutations could potentially modulate the conformation of the entire γ-secretase complex, which incorporates three critical components (*NCSTN*, *APH1*, and *PEN2*) in addition to *PSEN1* or *PSEN2*. Currently, no available software can analyze the 3D structure of multiprotein complexes like γ-secretase.

Among all AD cases, familial EOAD accounts for only approximately 1%, and over 80% of EOAD cases result from autosomal dominant missense mutations in either *APP*, *PSEN1,* or *PSEN2*. Notably, the *PSEN1* or *PSEN2* proteins are critical subunits of the γ-secretase complex.^18,19^ *PSEN1* is the most common cause of EOAD (>300 mutations have been reported), while *PSEN2* mutations are rare and often of unknown pathogenic significance (Alzgene database; http://www.alzforum.org/mutations).^19,20^ Both genes are also associated with other diseases, such as frontotemporal dementia and dilated cardiomyopathy (https://www.preventiongenetics.com/testInfo?val=6921).^19^ *PSEN2* mutations in particular have a wide range of age of onset (from 39 to 83 years) with a mean age of 55.3 years and over 50% above 60 years.^21^ All three patients we presented had symptom onset in their 50s and primarily amnestic presentation. However, Patient A had a relatively younger onset and more rapid cognitive and functional decline, along with a greater language involvement. All three patients and one of their asymptomatic siblings had a p.Ile120Ser substitution in *PSEN2*, a variant that has not yet been reported in Medline and the Alzgene genetic database (https://www.alzforum.org/mutations/psen-2).

## Conclusion

Investigation of the genetic bases of AD can help to confirm the diagnosis and aid in future care planning for family members who may choose to identify their own clinical risk.^22^ With the advent of disease-modifying therapies in AD, identifying novel genetic mutations will be increasingly important for monitoring disease response in genetic subtypes of AD, along with identifying at-risk family members to facilitate earlier treatment interventions. Asymptomatic patients with confirmed genetic mutations may also participate in appropriate clinical trials should they wish to. With this report, we aim to add to the literature on rare *PSEN2* variants causing familial EOAD and assist clinicians in providing appropriate genetic counseling. Furthermore, our case study has helped to confirm the eligibility of new participants (carriers of *PSEN2,* p.Ile120Ser) in an ongoing clinical trial, named the Dominantly Inherited Alzheimer Network (DIAN; http://clinicaltrials.gov/ct2/show/NCT01760005), which is focused on carriers of pathogenic *APP*, *PSEN1*, or *PSEN2* mutations with the goal of assessing the tolerability and biomarker efficacy of EOAD treatments.

## Supplemental Material

sj-docx-1-alr-10.1177_25424823251348676 - Supplemental material for A novel *PSEN2* mutation in amnestic early-onset Alzheimer's disease (EOAD): A familial case series Supplemental material, sj-docx-1-alr-10.1177_25424823251348676 for A novel *PSEN2* mutation in amnestic early-onset Alzheimer's disease (EOAD): A familial case series by Carl Froilan D Leochico, Ekaterina Rogaeva, Ljubica Zotovic, Ana Luiza Pinto Oliveira, Tina Le, Amit Singnurkar, Mario Masellis and Sara B Mitchell in Journal of Alzheimer's Disease Reports
