# Supplementary material for: A novel PSEN2 mutation in amnestic early-onset Alzheimer's disease (EOAD): A familial case series
Source: J Alzheimers Dis Rep. 2025 Jun 25;9:25424823251348676. doi: 10.1177/25424823251348676 (PMC12198541; doi:10.1177/25424823251348676)
Supplement: sj-docx-1-alr-10.1177_25424823251348676 - Supplemental material for A novel PSEN2 mutation in amnestic early-onset Alzheimer's disease (EOAD): A familial case series [file sj-docx-1-alr-10.1177_25424823251348676.docx]

**Supplemental Material**

**A novel PSEN2 mutation in amnestic early-onset Alzheimer’s disease (EOAD):**

**A familial case series**

**Supplemental Material 1: Genes analyzed**

**Patient A**

*ALS2, ANG, APOE, APP, ARSA, ATL1, ATP7B, BSCL2, CHCHD10, CHMP2B, CP, CSF1R, DCTN1, ERBB4, FIG4, FTL, FUS, GRN, HEXA, HNRNPA1, HSPD1, ITM2B, KIF5A, MAPT, MATR3, NEFH, NOTCH3, NPC1, OPTN, PANK2, PFN1, PRNP, PRPH, PSEN1, PSEN2, REEP1, SETX, SIGMAR1, SLC52A3, SNCA, SOD1, SORL1, SPAST, SPG11, SQSTM1, TARDBP, TBK1, TREM2, TUBA4A, TYROBP, UBE3A, UBQLN2, VAPB, VCP, WASHC5.*

**Patient B**

*APP*, *CHCHD10*, *CHMP2B*, *CSF1R*, *DCTN1*, *FUS*, *GRN*, *ITM2B*, *MAPT*, *PSEN1*, *PSEN2*, *SQSTM1*, *TARDBP*, *TBK1*, *TREM2*, *UBQLN2*, *VCP*.

**Patient C**

*APP*, *PSEN1*, *PSEN2.*
